# Muscle power-related parameters in middle-aged and older Brazilian women: a cross-sectional study

**DOI:** 10.1038/s41598-023-39182-7

**Published:** 2023-08-14

**Authors:** Hélio José Coelho-Júnior, Ivan de Oliveira Gonçalves, Francesco Landi, Riccardo Calvani, Matteo Tosato, Anna Picca, Emanuele Marzetti

**Affiliations:** 1 Department of Geriatrics, Orthopedics and Rheumatology, Center for Geriatric Medicine (Ce.M.I.), Università Cattolica del Sacro Cuore, Largo Francesco Vito 1, 00168 Rome, Italy; 2 Department of Health, Piaget University, Av. Mogi das Cruzes 1001, 08673-010, Suzano – SP, Brazil; 3 Fondazione Policlinico Universitario “Agostino Gemelli” IRCCS, Largo Agostino Gemelli 8, 00168, Rome, Italy; 4 Department of Medicine and Surgery, LUM University, SS100 km 18, 70010, Casamassima, Italy

**Keywords:** Signs and symptoms, Health care, Diagnosis, Disease prevention, Geriatrics

## Abstract

The present study was conducted to provide normative values for lower-limb muscle power estimated through equations based on the 5 times sit-to-stand (5STS) test in Brazilian older women. In addition, we investigated the association between muscle power parameters and age. The study followed a cross-sectional design. Participants were community-dwelling women. Candidates were considered eligible if they were 18 years or older, lived independently, and possessed sufficient physical and cognitive abilities to perform all measurements required by the protocol. The 5STS test was performed as fast as possible using a standard protocol. Absolute, relative, and allometric muscle power measures were estimated using 5STS-based equations. Two thousand four-hundred seventy-one women participated in the present study. Results indicated that muscle power-related parameters decreased linearly with age. Women 60–69 years showed a marginal reduction in absolute (− 5.2%), relative (− 7.9%), and allometric (− 4.0%) muscle power. A larger reduction was observed in those 70–79 years and reached ¼ of loss in participants ≥ 80, in comparison to middle-aged participants. Pearson’s correlation and linear regression analyses indicated that power-related parameters were negatively associated with age. In conclusion, data of the present study provide normative values for lower-limb muscle power parameters according to 5STS-based equations. We observed that muscle power-related parameters declined with age, such that participants 60–69, 70–79, and ≥ 80 years displayed lower absolute and relative muscle power compared middle-aged women. A later decline was observed in allometric muscle power. Relative muscle power declined to a greater extent than other parameters, suggesting a possible window of opportunity for interventions.

## Introduction

Physical performance is a multifaceted construct that involves motor tasks that allow interface between an individual and the environment^1–3^. Physical function commonly increases during childhood, remains substantially stable in adulthood, and decreases significantly past the fifth decade of life^1–3^. This scenario is especially concerning in older adults, given that a reduction in physical performance increases the risk of numerous negative outcomes^4–7^. Regarding sex-specific differences in physical function, women have lower physical performance than men^1–3,8^, higher prevalence of functional problems^9^ and disability^10^, and are at a greater risk of losing independence in daily activities due to impairments in physical function^9^.

Muscle power refers to the capacity to produce strength as fast as possible^11,12^. It has long been known as an important physical capacity for sports performance^13–15^. A growing number of studies have observed that muscle power declines earlier and faster with age than other important physical performance parameters (e.g., muscle strength)^8,16,17^. Furthermore, it might predict physical independence, functional performance, and mobility disability during old age^8,16–18^. More recently, it has been observed that muscle power is critical to the maintenance of functionality^19^. These premises led experts in the field to suggest that muscle power should be actively monitored during aging^11,12^.

The assessment of muscle power is based on laboratory tests (e.g., computer-interfaced pneumatic resistance machine) that are not completely adapted to the old population^20^. The existing tests also lack standardized protocols, have high costs, and are possibly associated with an increased risk of adverse events^20^. Such a scenario hampers the clinical assessment of muscle power in older adults. Recently, Alcazar et al.^21^ validated an easy-to-apply equation to estimate lower-limb muscle power using the time to complete the 5-time sit-to-stand test (5STS), chair's height, and the test person’s body mass and height. 5STS-based muscle power values are associated with numerous health aspects, including dynapenia, mobility problems, cognitive decline, frailty, disability, and low quality of life^22–24^. This approach may therefore be proposed as a feasible alternative to estimate lower-limb muscle power in clinical settings. A few studies have provided normative values according to Alcazar’s equation^22–24^. Available evidence includes data exclusively based on older adults^24^ or combined different populations^23^. Furthermore, only one study examined South American people^24^.

The projections of demographic transition in Latin America indicate a dramatic shift in population age structure^25^. Brazil is expected to have one of the largest old populations in Latin America by 2050, with almost 30% of citizens 60+ years^26^. Hence, the availability of normative values of muscle power across ages based on Alcazar et al.^21^ equations might represent a feasible evidence-based low-cost instrument for monitoring older adults and identify those at risk of negative events.

Based on these premises, the present study was designed to provide normative values for lower-limb muscle power using the 5STS equation^21^ in a large sample of middle-aged and older Brazilian women. In addition, we investigated the association between muscle power parameters and age.

## Results

Two thousand four-hundred seventy-one women participated in the study. The main characteristics and power-related parameters of study participants are shown in Table 1. Women 60–69 years had greater body mass than those in the ≤ 59 years group. Absolute and relative muscle power was lower in participants 60–69, 70–79 and 80+ years compared with those in the youngest groups (≤ 59 and 60–69 years). Allometric muscle power was lower in women 70–79 and 80+ years relative to participants 60–69 years. In addition, women 70–79 years had lower muscle power-parameters than those 60–69 years. No significant differences were observed between participants in the oldest groups (70–79 and 80+ years).

**Table 1:** Main characteristics of study participants (n = 2471). Data are shown as mean ±standard deviation or absolute numbers (%).

Variables	Age groups (years)			
	≤ 59 (n = 652)	60–69 (n = 622)	70–79 (n = 1027)	80+ (n = 170)
Age (years)	56.0 ± 2.4	63.9 ± 3.1a	74.0 ± 2.5ab	82.4 ± 3.9abc
Body mass (kg)	68.6 ± 15.0	71.5 ± 14.9a	70.8 ± 15.8	70.4 ± 15.1
Height (m)	1.57 ± 0.6	1.57 ± 0.6	1.57 ± 0.6	1.57 ± 0.5
BMI (kg/m^2^)	27.6 ± 5.7	28.8 ± 5.9a	28.5 ± 6.1	28.3 ± 5.8
Absolute muscle power (W)	203.8 ± 806.8	193.1 ± 646.6a	172.3 ± 576.8ab	164.9 ± 627.1ab
Relative muscle power (W/kg)	2.9 ± 0.8	2.6 ± 0.6a	2.4 ± 0.7ab	2.3 ± 0.7ab
Allometric muscle power (W/m^2^)	81.4 ± 30.5	78.1 ± 24.5	69.3 ± 22.4ab	67.9 ± 23.7ab
Hypertension (n, %)	326 (50.0)	311 (50.0)	414 (40.3)	47 (27.6)
Type II diabetes (n, %)	236 (36.2)	174 (28.0)	257 (25.0)	22 (13.0)
Osteoarthritis (n, %)	261 (40.0)	369 (59.3)	726 (70.7)	123 (72.4)
Cardiovascular (n, %)	39 (6.0)	50 (8.0)	158 (15.4)	14 (8.0)

*BMI* body mass index.

^a^*P* < 0.05 versus ≤ 59 years group.

^b^*P* < 0.05 versus 60–69 years group

^c^*P* < 0.05 versus 70–79 years group.

Table 2 shows absolute and relative differences in muscle power-related parameters. Muscle power parameters decreased linearly with age. Women 60–69 years showed a marginal reduction in absolute (− 5.2%), relative (− 7.9%), and allometric (− 4.0%) muscle power. A larger reduction was observed in those 70–79 years and reached ¼ of loss in the oldest participants (≥ 80 years) in comparison to middle-aged participants, except for allometric muscle power (16.5%). A mean decline rate of 13.2, 15.0, and 11.7% was observed for absolute, relative, and allometric muscle power, respectively.

**Table 2:** Absolute and relative variations in power-related parameters (n = 2471). Values are presented as absolute (relative) changes in power-related parameters.

Variables	Age groups (years)		
	60–69 (n = 622)	70–79 (n = 1027)	80+ (n = 170)
Absolute muscle power (W)	− 10.7 (− 5.2%)	− 31.5 (− 15.4%)	− 38.9 (− 19.1%)
Relative muscle power (W/kg)	− 0.23 (− 7.9%)	− 0.49 (− 16.8%)	− 0.59 (− 20.3%)
Allometric muscle power (W/m^2^)	− 3.3 (− 4.0%)	− 12.1 (− 14.8%)	− 13.5 (− 16.5%)

**Figure 1:**
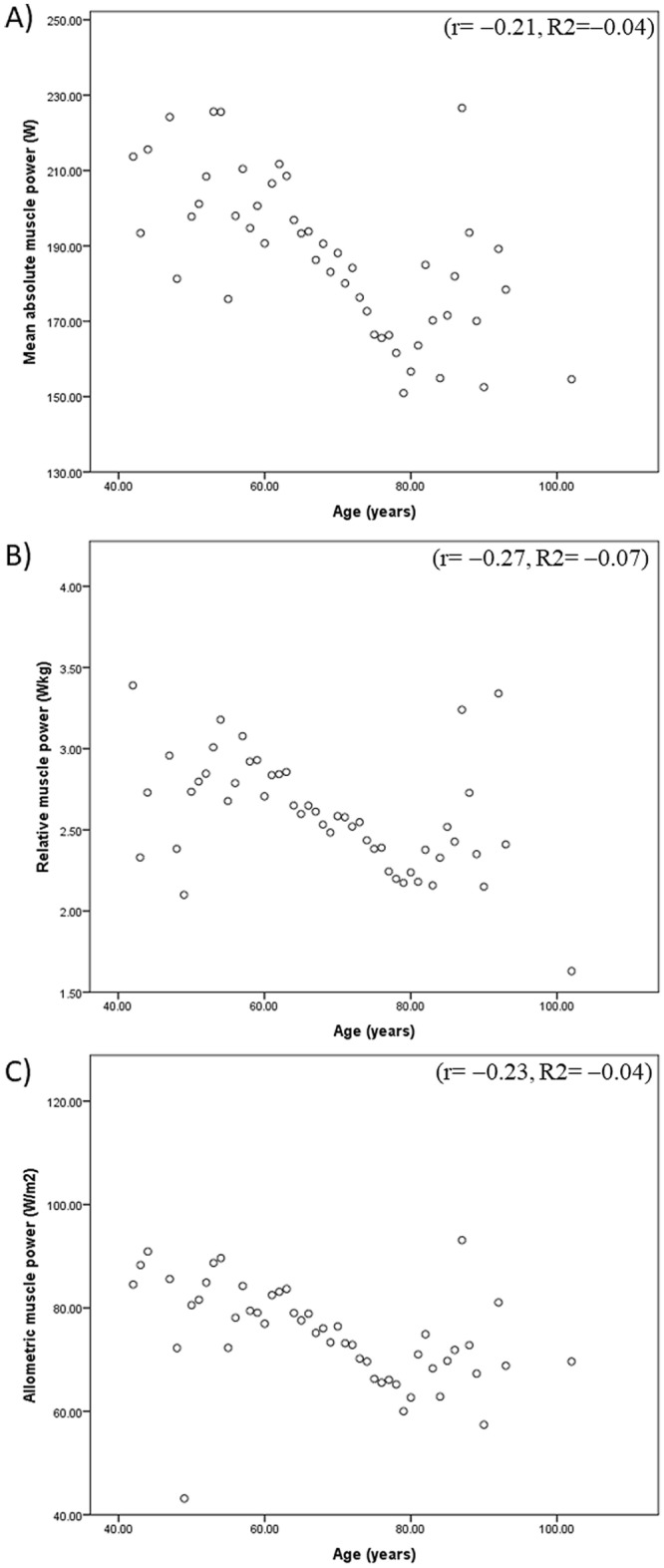
Person’s correlation for the association between age and (**A**) absolute, (**B**) relative, and (**C**) allometric muscle power.

Pearson’s correlation and linear regression analysis of the relationship between age and muscle power parameters are shown in Fig. 1. Pearson’s correlation indicated that age was weakly, negatively, and significantly correlated with absolute (r =  − 0.21), relative (r =  − 0.27), and allometric (r =  − 0.23) muscle power measures. According to the linear regression, absolute (R^2^ =  − 0.04, 95% confidence interval [CI] =  − 0.03, − 0.02, *P* < 0.001), relative (R^2^ =  − 0.07, 95% CI =  − 3.73, − 2.88, *P* < 0.001), and allometric muscle power measures (R^2^ =  − 0.04%, 95% CI =  − 0.08, − 0.06, *P* < 0.001) were negatively and significantly associated with age.

Normative values for absolute, relative, and allometric muscle power stratified by age groups are listed in Tables 3.

**Table 3:** Normative values for estimated absolute muscle power, stratified by age groups.

Age groups (years)	Observations (*n*)	Centiles					Mean (SD)
		5th	25th	50th	75th	95th	
		Muscle power (W)					
≤ 59	697	87.5	156.9	202.9	243.1	315.1	203.8 (06.8)
60–69	770	98.0	146.8	191.4	235.3	308.8	193.1 (646.6)
70–79	1051	83.8	128.2	168.4	212.3	277.7	172.3 (576.8)
≥ 80	202	84.7	129.0	159.9	204.3	273.3	164.9 (627.1)
	Relative muscle power (W/kg)						
≤ 59	697	1.8	2.4	2.8	3.3	4.2	2.9 (0.8)
60–69	770	1.5	2.1	2.6	3.1	4.0	2.6 (0.6)
70–79	1051	1.4	1.9	2.3	2.8	3.5	2.4 (0.7)
≥ 80	202	1.4	1.8	2.2	2.6	3.3	2.3 (0.7)
	Allometric muscle power (W/m^2^)						
≤ 59	697	39.9	64.2	79.9	96.9	123.2	81.4 (30.5)
60–69	770	41.3	60.2	77.0	93.1	120.7	78.1 (24.5)
70–79	1051	36.1	52.5	66.8	83.7	109.9	69.3 (22.4)
≥ 80	202	37.1	51.5	64.4	79.6	115.7	67.9 (23.7)

**Figure 2:**
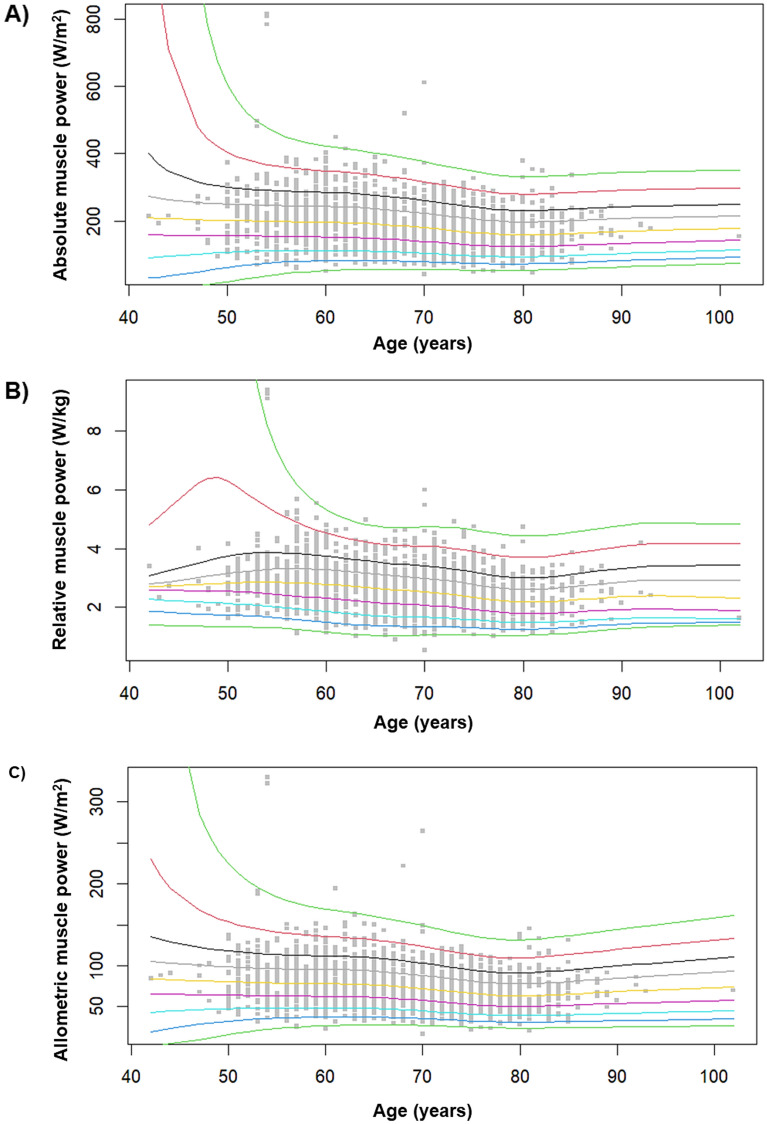
Reference percentiles for (**A**) absolute, (**B**) relative, and (**C**) allometric muscle power.

For each muscle power parameter, mean values ± SD and the 5th, 25th, 50th, 75th, and 95th percentiles are reported. Reference percentiles for muscle power measures are also depicted as charts in Fig. 2 to facilitate their practical implementation.

## Discussion

The main findings of the present study indicate that muscle power parameters are significantly associated with aging in Brazilian older women. Specifically, participants 60–69, 70–79, and 80+ years displayed reduced absolute and relative muscle power in comparison to middle-aged women. A significant decline in allometric muscle power was observed later in life, from the seventh decade. We also noted a greater decline in relative muscle power in comparison to other parameters, suggesting a possible target for interventions.

Our results are partially supported by prior studies that reported an age-related decline in muscle power. Lauretani et al.^8^ examined Italian older adults and found a linear association between age and lower-limb muscle power. Suetta et al.^27^ reported that power-related parameters started to decline in the fifth decade of life in Danish people. Similar findings were observed by Alcazar et al.^28^ in Spanish adults. Although these results are encouraging, they were obtained through laboratory-based tests.

A few studies examined the association between age and muscle power estimated using 5STS-based equations. In line with our findings, these investigations reported reductions in muscle power according to age^22^. However, sample characteristics differed from our study. Ramírez-Vélez et al.^24^ examined participants with lower average relative muscle power than those of the present study. When percentile values were compared, Colombians in the extremely high percentile (97th) had comparable values to those in the 50^th^ percentile of the present study. In contrast, Alcazar et al.^23^ examined a large cohort of European older adults with relatively greater percentiles for relative and allometric muscle power.

Cut-off values for muscle power parameters have been proposed. Baltasar-Fernandez et al.^22^ indicated that a cut-off value of ≤ 1.9 W/kg should be used to detect women at risk of frailty and impaired physical function. In their study, 45% of participants had values below this cut-off. Alcazar et al.^23^ proposed 2.1 W/kg and 61.5 W/m^2^ as cut-off values for relative and allometric muscle power, respectively, to identify older adults with mobility limitations. According to the cut-off points proposed by Baltasar-Fernandez et al.^22^ and Alcazar et al.^23^, 18.4% (n = 454) and 28.0% (n = 693) participants of the present study had low relative muscle power values, respectively. This suggests that region- and cultural-based cut-off values for muscle power parameters are necessary to properly identify older adults at risk of negative health-related events.

Results of Pearson’s correlation and linear regression suggest that variables other than chronological age impact the observed variations in muscle power parameters. When a complementary analysis adjusting the results according to the presence of hypertension (HTN), type II diabetes mellitus (T2DM), osteoarthritis (OA), and cardiovascular diseases (CVD) was performed (supplementary Table 1), age was only independently associated with muscle power parameters in women 70–79 years and with relative muscle power in those 60–69 and 80+ years. Although further analysis is needed, these findings indicate a possible effect of chronic conditions^29–32^ and pharmacological treatments^33,34^ on the associations between muscle power and age.

For instance, people with HTN might experience worse mobility and balance performances in comparison to normotensive counterparts^31,32^. The progression of T2DM leads to peripheral and autonomic nerve damage, which impacts lower-limb muscles and vision, thereby interfering with movement capacity^30^. OA involves pain, joint stiffness, and reduced range of motion^29^. Limited physical function and reductions in the capacity to perform activities of daily living are commonly observed in older adults with OA^35^. Regarding CVD, numerous studies have observed that stroke, myocardial infarction, and heart failure predispose to the development of other conditions associated with physical decline (e.g., sarcopenia)^36,37^.

Sarcopenia^38^, a neuromuscular disease characterized by the combination of reduced muscle strength and low muscle mass, and frailty^39^, a clinical condition triggered by a multisystem physiological derangement that results in a reduced ability to restore homeostasis after a stressful event, are frequently observed in advanced age. Both conditions are characterized by significant reductions in muscle strength and function and recognize disability as a common outcome^38,39^. Hence, the possibility that the presence of sarcopenia and frailty might have impacted our results cannot be ruled out.

Relative muscle power declined to a greater extension than other power-related parameters. Such declines were accompanied by significant increases in body mass and body mass index (BMI). This scenario might be explained, at least partly, by alterations in the hypothalamic—pituitary axis that occur during menopause and involve substantial reductions in energy expenditure and deposition of intra-abdominal fat^40,41^. Indeed, significant gains in fat, and consequently increases in BMI, occur for approximately 6 years after the final menstrual period, after which it stabilizes^40^.

Several social aspects with important influence on body composition undergo modifications at the beginning of old age. Retired women, for example, typically experience a 5% gain in body mass in the first two years of retirement compared with those who continue to work at least 20 h per week^42^. These findings suggest that early during old age women pass through important changes in body composition that might affect their physical performance. On the other hand, reductions in absolute muscle power might reflect an age-related decline in neuromuscular function (e.g., neuromuscular junction)^43^ and structure (e.g., reduction in alpha-motoneurons)^44^, in addition to being associated with muscle atrophy and dynapenia^1,2,8^.

Another important result of the present study is that older women 80+ years experienced a decline of ~ 25% in muscle power-related parameters in comparison to middle-aged participants. These results are concerning and indicate that very old women require special attention to prevent losses in muscle power. The mechanisms underlying this pattern of changes are beyond the scope of our study. However, several aspects have repeatedly been observed in very old adults that might help to explain these findings, including chronic pain^45^, mental distress^46^, sedentary behavior^47,48^, and protein intake^49^.

The current study offers normative values by which Brazilian older women might be monitored. Results also provide evidence to compare muscle power parameters in older adults from different geographic regions. Moreover, we observed two timeframes when strategies appear to be necessary to prevent or at least limit substantial losses in muscle power. Specifically, the beginning of old age is accompanied by important gains in body mass, suggesting that specific diet patterns, psychosocial support, and physical activity recommendations might be required. In contrast, very old adults might require a more complex approach. A recent large multicentric randomized clinical trial observed that a 24-month intervention based on physical activity plus personalized nutritional counseling improved physical performance in older adults with functional limitations^50^.

Resistance training, the type of exercise in which muscle contractions occur to maintain or move a load^51^, is a widely accepted strategy to improve muscle power in people with different conditions^52,53^, including frail older adults^54^. Experts in the field have mentioned that resistance training should include muscle concentric contractions performed as fast as possible (i.e., explosive resistance training) to produce optimal gains in muscle power and functional improvements^52^, likely avoiding disability^12^. Numerous exercise protocols including different configurations (set, volume, rest interval) and using machines, elastic bands, or body mass have been tested, demonstrated effectiveness, and might be easily reproduced in clinical practice^55,56^. Health professionals responsible for exercise prescription should adequate the exercise program according to women’s age, health status, and expected goals.

Our study is not free of limitations. First, specific muscle power, adjusted according to muscle mass, was not estimated in the present study and the possibility that muscle atrophy influenced our results cannot be ruled out. Second, older women were not screened for sarcopenia^38^ or frailty^39^. Third, important information associated with the presence of chronic conditions, such as pharmacological therapy and disease status, was not recorded. Fourth, hormonal levels were not assessed. Fifth, only community-dwelling women were examined, and extrapolations to men or people in other contexts (e.g., institutionalized) should be made with caution. Sixth, correlation analysis was not corrected according to numerous covariables that might influence muscle power, including physical activity levels, diet quality, and sleep. Seventh, normative values were not tested against health-related events. Finally, the results shown in this work are derived from cross-sectional observations. The possibility cannot be ruled out that differences in birth cohorts may have influenced some of the assessed parameters. A deeper understanding of age-dependent differences in muscle power requires an analysis of prospective data that are unavailable at this stage for our study.

In conclusion, data of the present study provide normative values for lower-limb muscle power parameters according to 5STS-based equations. We observed that muscle power-related parameters declined with age, such that participants 60–69, 70–79, and ≥ 80 years showed reduced absolute, relative, and allometric muscle power compared with middle-aged women. Finally, we observed that relative muscle power declined to a greater extent than other parameters, suggesting a possible target for interventions.

## Methods

This study used a large-scale cross-sectional design and was approved by the Research Ethics Committee of the University of Mogi das Cruzes (UMC, São Paulo, Brazil). All study procedures were conducted in compliance with the Declaration of Helsinki and the Resolution 196/96 of the National Health Council.

### Participants

Participants were recruited between January 2015 and January 2018 in a community senior center located in the metropolitan area of São Paulo, Brazil. The study was advertised through posters placed in public sites (e.g., parks, city hall, public offices, bus stops, train stations), local radios, and newspapers. People were also invited to participate by direct contact by the research team. Candidate participants were eligible if they were 18 years or older, lived independently, and possessed physical and cognitive abilities to perform the 5STS test. All participants provided written informed consent prior to inclusion.

### Anthropometric measurements

An analog weight scale with a stadiometer (Filizola, Brazil) was used to measure body mass and height. The BMI was calculated as the ratio between body mass (kg) and the square of height (m^2^).

### Five-time sit-to-stand test

The 5STS test was administered by two experienced exercise physiologists in a dedicated room within the senior center. One examiner was responsible for detailing the operational procedures, demonstrating the test before the assessment, quantifying performance, and evaluating motor patterns. The other examiner ensured participant safety by providing occasional verbal and/or tactile cueing, if needed, without interfering with the physical function test. After the explanation and before testing, participants performed a familiarization trial to ensure they had fully understood the test. All tests were performed twice, and the best result was used for the analysis.

The test involved rising from a chair 5 times as quickly as possible with arms folded across the chest. Timing began when participants raised their buttocks off the chair and was stopped when they were seated at the end of the fifth stand^2^. Time performance was quantified using a stopwatch (Vollo Sports, São Paulo, Brazil). The test reliability in the present study was higher than 0.8 (κ = 0.97).

Absolute, relative (adjusted by body mass), and allometric (adjusted by height) muscle power values were estimated according to the equations proposed by Alcazar et al.^21^:

- $${\text{Absolute}}\;{\text{muscle}}\,{\text{power}}:{ }{\raise0.7ex\hbox{${\left[ {{\text{Body}}\,{\text{mass (kg) }}{\times}\,0.9\;{\times}\,{\text{g }}{\times}\left( {{\text{height (m)}}\,{\times}\,0.5{ }\;{\text{-}}\;{\text{chair}}\,{\text{height (m)}}} \right)} \right]}$} \!\mathord{\left/ {\vphantom {{\left[ {{\text{Body}}\,{\text{mass }}0.9\;{\text{g }}\left( {{\text{height}}\,0.5{ }\;{\text{chair}}\,{\text{height}}} \right)} \right]} {5{\text{STS }}\left( {\text{s}} \right)\;{ }0.1}}}\right.\kern-0pt} \!\lower0.7ex\hbox{${5{\text{STS }}\left( {\text{s}} \right){\times}{ }\,0.1}$}}$$
- $${\text{Relative}}\;{\text{muscle}}\;{\text{power}}:{\text{Absolute}}\;{\text{muscle}}\;{\text{power }}\left( {\text{W}} \right)/{\text{kg}}$$
- $${\text{Allometric}}\;{\text{muscle}}\;{\text{power}}:{\text{Absolute}}\;{\text{muscle}}\,{\text{power }}\left( {\text{W}} \right)/{\text{m}}^{{2}}$$

### Disease conditions

Information pertaining to disease conditions was collected through self-report and careful review of medical charts of the community senior center.

### Statistical analysis

Data were not normally distributed. Non-Gaussian distribution might be ignored if large sample sizes (> 30–40 participants) with values representative of a “real population” are investigated^57,58^. Continuous variables are expressed as mean ± standard deviation (SD) or absolute numbers (percentage). Differences in continuous variables among groups (i.e., ≤ 59, 60–69, 70–79, 80+) were assessed via one-way analysis of variance (ANOVA). When appropriate, Bonferroni post hoc analyses were performed to determine whether there were significant differences between groups. Posttests were performed to investigate linear trends of decline in power-related parameters in relation to age. Pearson's correlations were used to explore the relationship between muscle power and age. Coefficients were classified as: negligible (0.00–0.10), weak (0.10–0.39), moderate (0.40–0.69), strong (0.70–0.89), and very strong (0.90–1.00)^59^. Linear regression analysis was used to test the associations between age and muscle power-related parameters. Confidence intervals (CIs) that included the number of 1 were not statistically significant. Significance was set at 5% (*P* < 0.05) for all tests. All analyses were performed using the SPSS software (version 23.0, SPSS Inc., Chicago, IL). Smoothed percentile curves for absolute muscle power values were constructed using the lambda-mu-sigma (LMS) method (LMS Chart Maker Pro Version 2.54, Medical Research Council, London, UK), as described elsewhere^1^.

### Supplementary Information

Supplementary Information.

## Data Availability

The datasets generated during and/or analysed during the current study are available from the corresponding author on reasonable request.

## References

[1] Landi F (2020). Normative values of muscle strength across ages in a ‘real world’ population: Results from the longevity check-up 7+ project. J. Cachexia Sarcopenia Muscle.

[2] Coelho-Junior HJ (2021). Age- and gender-related changes in physical function in community-dwelling Brazilian adults aged 50 to 102 years. J. Geriatr. Phys. Therapy.

[3] Marzetti E (2018). Age-related changes of skeletal muscle mass and strength among Italian and Taiwanese older people: Results from the Milan EXPO 2015 survey and the I-Lan Longitudinal Aging Study. Exp. Gerontol..

[4] Rosano C (2005). Association between physical and cognitive function in healthy elderly: The health, aging and body composition study. Neuroepidemiology.

[5] Abellan van Kan G (2009). Gait speed at usual pace as a predictor of adverse outcomes in community-dwelling older people an International Academy on Nutrition and Aging (IANA) Task Force. J. Nutr. Health Aging.

[6] Studenski S (2011). Gait speed and survival in older adults. JAMA.

[7] Pavasini R (2016). Short Physical Performance Battery and all-cause mortality: systematic review and meta-analysis. BMC Med..

[8] Lauretani F (2003). Age-associated changes in skeletal muscles and their effect on mobility: An operational diagnosis of sarcopenia. J. Appl. Physiol..

[9] Crimmins EM, Hayward MD, Saito Y (1996). Differentials in active life expectancy in the older population of the United States. J. Gerontol. B Psychol. Sci. Soc. Sci..

[10] Leveille, S. G., Penninx, B. W. J. H., Melzer, D., Izmirlian, G. & Guralnik, J. M. Sex differences in the prevalence of mobility disability in old age: The dynamics of incidence, recovery, and mortality. *J. Gerontol. B Psychol. Sci. Soc. Sci.* **55** (2000). 10.1093/geronb/55.1.s41 10728129

[11] Cadore EL, Izquierdo M (2018). Muscle power training: A hallmark for muscle function retaining in frail clinical setting. J. Am. Med. Dir. Assoc..

[12] Izquierdo M, Cadore EL (2014). Muscle power training in the institutionalized frail: A new approach to counteracting functional declines and very late-life disability. Curr. Med. Res. Opin..

[13] Kraemer WJ, Looney DP (2012). Underlying mechanisms and physiology of muscular power. Strength Cond. J..

[14] Haff GG, Nimphius S (2012). Training principles for power. Strength Cond. J..

[15] Haff G, Triplett T (2005). Essentials of Strength Training and Conditioning.

[16] Suzuki T, Bean JF, Fielding RA (2001). Muscle power of the ankle flexors predicts functional performance in community-dwelling older women. J. Am. Geriatr. Soc..

[17] Bean JF (2003). A Comparison of leg power and leg strength within the InCHIANTI study: Which influences mobility more?. J. Gerontol. A Biol. Sci. Med. Sci..

[18] Hetherington-Rauth M (2022). Relative sit-to-stand muscle power predicts an older adult’s physical independence at age of 90 yrs beyond that of relative handgrip strength, physical activity, and sedentary time: A cross-sectional analysis. Am. J. Phys. Med. Rehabil..

[19] Alcazar J (2021). Threshold of relative muscle power required to rise from a chair and mobility limitations and disability in older adults. Med. Sci. Sports Exerc..

[20] Alcazar J, Guadalupe-Grau A, García-García FJ, Ara I, Alegre LM (2018). Skeletal muscle power measurement in older people: A systematic review of testing protocols and adverse events. J. Gerontol. A Biol. Sci. Med. Sci..

[21] Alcazar J (2018). The sit-to-stand muscle power test: An easy, inexpensive and portable procedure to assess muscle power in older people. Exp. Gerontol..

[22] Baltasar-Fernandez I (2021). Relative sit-to-stand power cut-off points and their association with negatives outcomes in older adults. Sci. Rep..

[23] Alcazar J (2021). Relative sit-to-stand power: Aging trajectories, functionally relevant cut-off points, and normative data in a large European cohort. J. Cachexia Sarcopenia Muscle.

[24] Ramírez-Vélez R (2022). Sit to stand muscle power reference values and their association with adverse events in Colombian older adults. Sci. Rep..

[25] Duda-Nyczak, M. Demographic transition and achieving the SDGs in Latin America and the Caribbean: A regional overview of the National Transfer Accounts (2021).

[26] World Health Organization—Percentage of total population aged 60 years or over. https://platform.who.int/data/maternal-newborn-child-adolescent-ageing/indicator-explorer-new/mca/percentage-of-total-population-aged-60-years-or-over.

[27] Suetta C (2019). The Copenhagen Sarcopenia Study: Lean mass, strength, power, and physical function in a Danish cohort aged 20–93 years. J. Cachexia Sarcopenia Muscle.

[28] Alcazar J (2020). Age- and sex-specific changes in lower-limb muscle power throughout the lifespan. J. Gerontol. A Biol. Sci. Med. Sci..

[29] Hunter DJ, Bierma-Zeinstra S (2019). Osteoarthritis. Lancet.

[30] Kirkman MS (2012). Diabetes in older adults. Diabetes Care.

[31] Junior HJC (2016). Hypertension and functional capacities in community-dwelling older women: A cross-sectional study. Blood Press..

[32] Hausdorff JM, Herman T, Baltadjieva R, Gurevich T, Giladi N (2003). Balance and gait in older adults with systemic hypertension. Am. J. Cardiol..

[33] Brink M (2001). Angiotensin II induces skeletal muscle wasting through enhanced protein degradation and down-regulates autocrine insulin-like growth factor I. Endocrinology.

[34] Yoshida T (2013). Molecular mechanisms and signaling pathways of angiotensin II-induced muscle wasting: Potential therapeutic targets for cardiac cachexia. Int. J. Biochem. Cell Biol..

[35] Hawker GA, King LK (2022). The burden of osteoarthritis in older adults. Clin. Geriatr. Med..

[36] Brum PC, Bacurau AV, Cunha TF, Bechara LRG, Moreira JBN (2014). Skeletal myopathy in heart failure: Effects of aerobic exercise training. Exp. Physiol..

[37] Coelho Junior, H. J. *et al.* Inflammatory mechanisms associated with skeletal muscle sequelae after stroke: Role of physical exercise. *Mediators Inflamm.* **2016** (2016). 10.1155/2016/3957958 PMC5018330 27647951

[38] Cruz-Jentoft AJ, Sayer AA (2019). Sarcopenia. Lancet.

[39] Hoogendijk EO (2019). Frailty: Implications for clinical practice and public health. Lancet.

[40] Greendale, G. A. *et al.* Changes in body composition and weight during the menopause transition. *JCI Insight* **4** (2019). 10.1172/jci.insight.124865 PMC6483504 30843880

[41] Davis SR (2012). Understanding weight gain at menopause. Climacteric.

[42] Forman-Hoffman VL (2008). Retirement and weight changes among men and women in the health and retirement study. J. Gerontol. B Psychol. Sci. Soc. Sci..

[43] Li L, Xiong W-C, Mei L (2018). Neuromuscular junction formation, aging, and disorders. Annu. Rev. Physiol..

[44] Tomlinson BE, Irving D (1977). The numbers of limb motor neurons in the human lumbosacral cord throughout life. J. Neurol. Sci..

[45] Wettstein M, Schilling OK, Wahl H-W (2022). Trajectories of pain in very old age: The role of eudaimonic wellbeing and personality. Front. Pain Res..

[46] Fässberg MM (2019). Epidemiology of suicidal feelings in an ageing Swedish population: From old to very old age in the Gothenburg H70 Birth Cohort Studies. Epidemiol. Psychiatr. Sci..

[47] Varesco G (2022). Association between physical activity, quadriceps muscle performance, and biological characteristics of very old men and women. J. Gerontol. A Biol. Sci. Med. Sci..

[48] Granic A (2019). Factors associated with change in self-reported physical activity in the very old: The Newcastle 85+ study. PLoS ONE.

[49] Mendonça N, Kingston A, Granic A, Jagger C (2019). Protein intake and transitions between frailty states and to death in very old adults: The Newcastle 85+ study. Age Ageing.

[50] Bernabei R (2022). Multicomponent intervention to prevent mobility disability in frail older adults: Randomised controlled trial (SPRINTT project). BMJ.

[51] Garber CE (2011). American College of Sports Medicine position stand. Quantity and quality of exercise for developing and maintaining cardiorespiratory, musculoskeletal, and neuromotor fitness in apparently healthy adults: Guidance for prescribing exercise. Med. Sci. Sports Exerc..

[52] Fragala MS (2019). Resistance training for older adults. J. Strength Cond. Res..

[53] Coelho-Júnior HJ (2021). Evidence-based recommendations for resistance and power training to prevent frailty in community-dwellers. Aging Clin. Exp. Res..

[54] Coelho-Júnior HJ, Uchida MC (2021). Effects of low-speed and high-speed resistance training programs on frailty status, physical performance, cognitive function, and blood pressure in prefrail and frail older adults. Front. Med..

[55] Balachandran AT (2022). Comparison of power training vs traditional strength training on physical function in older adults: A systematic review and meta-analysis. JAMA Netw. Open..

[56] Straight CR, Lindheimer JB, Brady AO, Dishman RK, Evans EM (2016). Effects of resistance training on lower-extremity muscle power in middle-aged and older adults: A systematic review and meta-analysis of randomized controlled trials. Sports Med..

[57] Ghasemi A, Zahediasl S (2012). Normality tests for statistical analysis: A guide for non-statisticians. Int. J. Endocrinol. Metab..

[58] Altman DG, Bland JM (1995). Statistics notes: The normal distribution. BMJ.

[59] Schober P, Schwarte LA (2018). Correlation coefficients: Appropriate use and interpretation. Anesth. Analg..

