# Supplementary material for: Muscle power-related parameters in middle-aged and older Brazilian women: a cross-sectional study
Source: Sci Rep. 2023 Aug 14;13:13186. doi: 10.1038/s41598-023-39182-7 (PMC10425341; doi:10.1038/s41598-023-39182-7)
Supplement: Supplementary file 1 — Supplementary Information. [file 41598_2023_39182_MOESM1_ESM.docx]

| **Table SM1.** Association between age and muscle power parameters adjusted by the presence of chronic conditions | | |
| --- | --- | --- |
|  | **β (95% CI)*** | P-value |
| ≤59 years | | |
| Absolute muscle power | -0.002 (-0.004, 0.001) | 0.174 |
| Relative Muscle power | 0.094 (-0.161, 0.349) | 0.468 |
| Allometric muscle power | -0.006 (-0.013, 0.001) | 0.104 |
|  | |  |
| 60-69 years | | |
| Absolute muscle power | -0.003 (-0.006, 0.000) | 0.068 |
| Relative Muscle power | -0.532 (-0.864, -0.261) | 0.000 |
| Allometric muscle power | -0.009 (-0.017, 0.000) | 0.058 |
|  |  |  |
| 70-79 years | | |
| Absolute muscle power | -0.007 (-0.009, -0.004) | 0.0001 |
| Relative Muscle power | -0.743 (-0.974, -0.511) | 0.0001 |
| Allometric muscle power | -0.020 (-0.027, -0.013) | 0.0001 |
|  |  |  |
| 80+ years | | |
| Absolute muscle power | 0.007 (0.009, 0.013) | 0.064 |
| Relative Muscle power | 0.952 (0.321, 1.584) | 0.003 |
| Allometric muscle power | 0.017 (-0.001, 0.036) | 0.060 |
| *Adjusted by hypertension, type II diabetes mellitus, osteoarthirts, and cardiovascular diseases | | |
